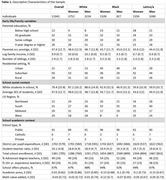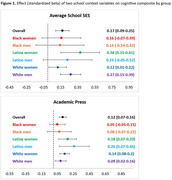# Cognition Four Decades After High School: Does School Context Matter for Cognitive Disparities at Midlife?

**DOI:** 10.1002/alz.091879

**Published:** 2025-01-09

**Authors:** Jennifer J. Manly, Koit Hung, Adam M. Brickman, Eric Grodsky, Chandra Muller, John Robert Warren, Michael Culbertson

**Affiliations:** ^1^ Taub Institute for Research on Alzheimer’s Disease and the Aging Brain, Columbia University, New York, NY USA; ^2^ University of Texas at Austin, Austin, TX USA; ^3^ University of Wisconsin ‐ Madison, Madison, WI USA; ^4^ University of Minnesota, Minneapolis, MN USA

## Abstract

**Background:**

Structural racism shapes educational quality by perpetuating unequal access to school resources, creating disproportionate exposure to disciplinary actions, and limiting access to advanced courses among racially minoritized children. School quality is linked to later life ADRD risk, but the benefit may vary across groups. We asked 1) whether the same high school social contexts and academic resources predict midlife cognitive functioning across race, ethnicity, and gender, and 2) how much could disparities in midlife cognitive function be narrowed if everyone had equal access to high quality high schools?

**Method:**

Data was from the nationally representative High School and Beyond cohort, which prospectively followed 12,530 Americans from high school through age ∼60 and administered telephone‐ and web‐based measures of memory, language, and attention in 2021‐22. Extensive information about family, high school social and academic context, and student achievements was gathered directly from students and their schools in 1980‐1982. OLS regression models estimated associations between specific school variables and an IRT‐derived cognitive composite, before and after adjustment for confounders in race, ethnicity, and sex stratified models.

**Result:**

Higher average SES of students in each high school predicted better midlife cognitive test scores among White people and Latina women, but not among Latino men or Black participants. This association weakened after adding school academic context variables. A composite measure capturing school‐level academic engagement and rigor (academic press) predicted better cognition among White and Latinx participants but not among Black participants. If everyone had access to high schools of the same quality as the group with the highest test scores (White women), and adjusting for family confounders, disparities between White women and participants from racially minoritized groups would be substantially reduced (by 9% among Black women, 5% among Black men, and 6% among Latino men and Latina women).

**Conclusion:**

Investment in high schools that serve Black and Latinx children could meaningfully narrow disparities in cognition four decades later, particularly among Black women. Later life cognitive benefit of specific aspects of school context, such as high SES peers and academic rigor, had differential benefit depending on race, ethnicity, and sex.